# Neurofilament light chain marks severity of papilledema in idiopathic intracranial hypertension

**DOI:** 10.1007/s10072-023-06616-z

**Published:** 2023-01-23

**Authors:** Theresia Knoche, Verena Gaus, Paula Haffner, Alexander Kowski

**Affiliations:** Dept. of Neurology, Charité – Universitätsmedizin Berlin – Campus Virchow Klinikum, Augustenburger Platz 1, 13353 Berlin, Germany

**Keywords:** Idiopathic intracranial hypertension, Neurofilament light chain, Papilledema, CSF opening pressure

## Abstract

**Background:**

Neurofilament light chain (NfL) reflects axonal damage in neurological disorders. It has recently been evaluated in idiopathic intracranial hypertension (IIH). A biomarker indicating the severity of optic nerve damage in IIH could support diagnostic accuracy and therapeutic decisions.

**Methods:**

We retrospectively reviewed NfL concentrations in the cerebrospinal fluid (CSF) of 35 IIH patients and 12 healthy controls, who had received diagnostic workup for IIH in our clinic. The diagnosis of IIH was made according to the modified Friedman criteria for IIH and for IIH without papilledema Friedman DI et al Neurol 81:1159–1165 (2013) [1]. NfL in the CSF (CSF-NfL) was correlated with the severity of papilledema and with CSF opening pressure.

**Results:**

CSF-NfL correlated with CSF opening pressure at the time of collection. In patients with IIH and moderate or severe papilledema, CSF-NfL was significantly increased compared to patients with mild or no papilledema. Healthy controls with raised intracranial pressure showed no relevant elevation of CSF-NfL.

**Conclusion:**

CSF-NfL appears to correlate with the severity of papilledema in IIH and with CSF opening pressure and may therefore be a predictor of optic nerve damage in IIH patients.

## Introduction

Idiopathic intracranial hypertension (IIH) is a rare headache syndrome mainly observed in overweight women of childbearing age [1–4]. The defining feature is an elevated intracranial pressure (ICP), without an identifiable source of hydrocephalus or cerebral mass lesions. The exact pathophysiology of IIH remains vague [5]. Patients report progressive, sometimes permanent visual impairment [6]. The diagnosis of IIH is based on the revised Friedman criteria, which demands the presence of papilledema for the diagnosis of definite IIH [7]. Optic nerve damage is the commonest complication in IIH [8, 9]. Its prevention is among the prior therapeutic targets [10]. The therapeutic strategy comprises reduction of intracranial pressure by medication and by weight loss in overweight patients. Although the severity of papilledema is associated with the extent of optic nerve damage, it evolves with delay, sometimes precedes symptoms, and can remain unnoticed for a certain time [11, 12]. In the later stages of the disease, optic nerve atrophy can even mask papilledema.

Neurofilament light chain (NfL) is a polypeptide that gives structural support to the axon and regulates its diameter. NfL marks axonal loss in the central nervous system in various neurodegenerative disorders [13, 14] and has been shown to predict visual outcome in optic neuritis [15]. Thus, NfL measurements could be of substantial benefit for identifying patients at risk of lasting visual impairment or of subclinical damage to the optic nerve in IIH. Recently, it has been demonstrated that NfL in the CSF (CSF-NfL) and in the serum relates to the extent of papilledema and corresponded with poor ophthalmological outcome in a cohort of 61 patients with suspected IIH [16]. Further studies on the subject are, to the best of our knowledge, not available. We hypothesize, however, that CSF-NfL reflects the extent of papilledema due to increased intracranial pressure.

## Methods

**Fig. 1:**
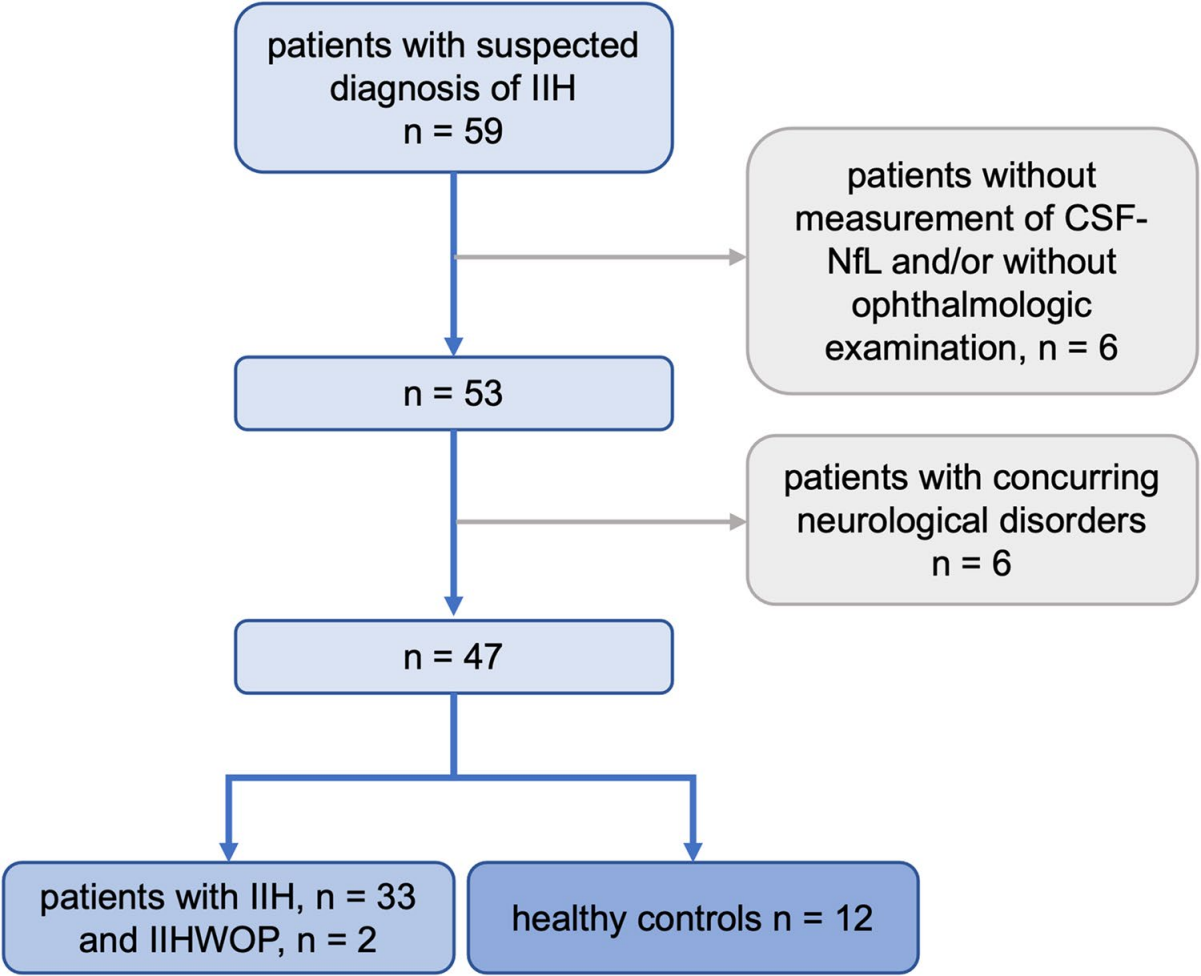
Flow-chart of patient selection. Out of 59 patients admitted to our hospital for suspected IIH, 6 were excluded because of incomplete diagnostic workup. Six were excluded because different neurological disorders were found. Among the 47 remaining patients, 35 met the Friedman diagnostic criteria for IIH/IIHWOP. The existence of IIH and other neurological disorders was ruled out in 12 patients, which were used as healthy controls

For this retrospective study, we reviewed medical charts of adult patients who had received diagnostic evaluation for suspected or already diagnosed IIH at our neurological department (Charité—Universitätsmedizin Berlin, Campus Virchow Klinikum) between January 2020 and June 2021. Only patients who had received ophthalmological and neurological examinations, including a lumbar puncture with CSF opening pressure measurement and measurement of CSF-NfL, were included. Diagnosis of IIH was made according to the modified Friedman criteria for IIH and for IIH without papilledema (IIHWOP) [7]. Patients with other neurological disorders were excluded from this study. A study flowchart of patients meeting inclusion and exclusion criteria can be found in Fig. 1. Patients who had received diagnostic workup for suspected IIH, including ophthalmological and neurological examinations as well as measurements of CSF-NfL and CSF opening pressure, were enrolled as healthy controls if, after the completion of the diagnostic process, no neurological disorders were found. Absolute CSF-NfL and ratios of CSF-NfL to age-adjusted reference intervals, because NfL levels increase with age, were documented. Serum NfL measurements were not available. The CSF opening pressure, as measured during lumbar puncture, was extracted from neurological reports. Fundoscopic papilledema grading was extracted from ophthalmologic reports. Grading was based on the modified Frisén scale [17]. Frisén grades for the most severely affected eyes were transformed into mild (grade 1), moderate (grades 2 and 3) and severe (grades 4 and 5) papilledema. The presence of optic nerve atrophy was noted. We statistically analysed CSF-NfL, the grade of papilledema, the CSF opening pressure and whether IIH was diagnosed.

This research study was conducted retrospectively from data obtained for clinical purposes. Retrospective data extraction and analysis was approved by the ethics committee of Charité – Universitätsmedizin Berlin (Application No. EA4/004/21).

### Statistical analysis

All statistical analyses were performed in SPSS 27 (IBM Corp. Released 2020. IBM SPSS Statistics for Windows, Version 27.0. Armonk, NY: IBM Corp). Continuous variables were described by the mean and standard deviation. Variables were checked for normal distribution using the Shapiro–Wilk test and Q-Q-plots. The CSF-NfL showed a right-skewed distribution and was log-transformed for subsequent analyses (log 10 = lg). CSF-NfL ratios were calculated from the quotient of the CSF-NfL and the age-adjusted normal value (CSF-NfL/age-adjusted normal value). For the statistical analysis, an unpaired *t*-test was performed for group comparisons of continuous variables and a chi-square test or Fisher’s exact test for group comparisons with regard to sex. Correlation between CSF-NfL ratios and the CSF opening pressure was ﻿tested for linear correlation using the Pearson correlation coefficient. A two-tailed *p*-value of < 0.05 was considered statistically significant.

## Results

We identified 59 patients with suspected IIH, of which 53 had received measurements of CSF-NfL and ophthalmological and neurological examinations. Six patients were excluded because alternative neurological diseases (such as meningeosis carcinomatosa and arteriovenous malformations) were found. The remaining 47 patients consisted of 35 with newly made or pre-existing diagnoses of IIH/IIHWOP (IIH: *n* = 33, IIHWOP: *n* = 2) and 12 patients, for which the suspected diagnosis of IIH was rejected and no other disease could be found, who are subsequently referred to as healthy controls. Five of the IIH/IIHWOP patients had unilateral abducens nerve palsy at the time of examination, three in the IIH-group and the two IIHWOP patients. Patients and healthy controls did not significantly differ with respect to age and sex. Detailed patient characteristics are given in Table 1.

**Table 1:** Demographic and clinical characteristics of all patients and the subgroups IIH/IIHWOP and healthy controls

	All patients (*n* = 47)	IIH/IIHWOP (*n* = 33/2)	Healthy controls (*n* = 12)	
Age (mean ± SD)	35.2 ± 11.1	35.8 ± 10.8	33.3 ± 12.1	*p* = 0.2
- Female	34 (72%)	26 (74%)	8 (67%)	*p* = 0.7
- Male	13 (28%)	9 (26%)	4 (33%)	
CSF-NfL (lg)/age-adjusted normal value (mean ± SD)	− 0.017 ± 0.32	0.04 ± 0.32	− 0.19 ± 0.29	*p* = 0.03
Absolute CSF-NfL [pg/ml] (mean ± SD)	563.8 ± 446.7	657.3 ± 481.9	291.1 ± 87.6	-
CSF-NfL ratios = CSF-NfL/age-adjusted normal value (mean ± SD)	1.2 ± 1.0	1.4 ± 1.1	0.8 ± 0.3	-
CSF opening pressure [cm H_2_O] (mean ± SD)	30.8 ± 9.3	33.1 ± 9.2	23.9 ± 5.9	-
Papilledema (*n*)				
- None	24 (51%)	12 (34%)	12 (100%)	
- Mild	7 (15%)	7 (20%)	-	
- Moderate	9 (19%)	9 (26%)	-	-
- Severe	7 (15%)	7 (20%)	-	
Abducens nerve palsy (*n*)	5 (10.6%)	5 (10.6%)	0	-

**Fig. 2:**
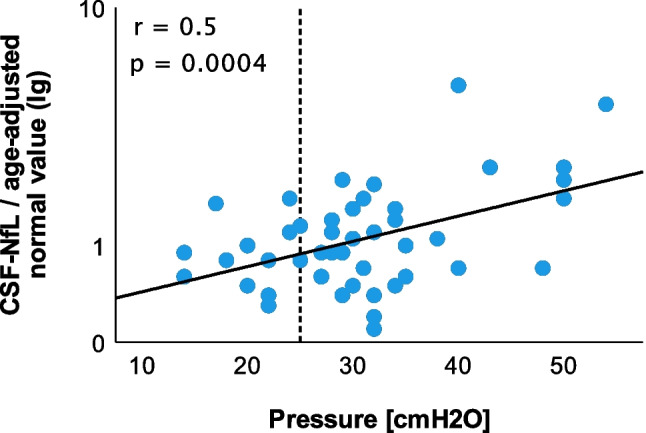
Correlation of CSF-NfL and CSF opening pressure of all patients (IIH/IIHWOP and healthy controls, Pearson correlation). The correlation coefficient of *r* = 0.50 indicates a moderate correlation of CSF-NfL ratios with CSF opening pressure. The vertical bar indicates the 25 cmH_2_O cut off for pathologically elevated CSF pressure

CSF-NfL ratios had a moderate correlation with CSF opening pressure (Fig. 2, *r* = 0.50, *p* = 0.0004) in all patients. CSF-NfL had a moderate correlation with age (*r* = 0.3; *p* = 0.02). CSF-NfL did not significantly differ with respect to sex.

**Fig. 3:**
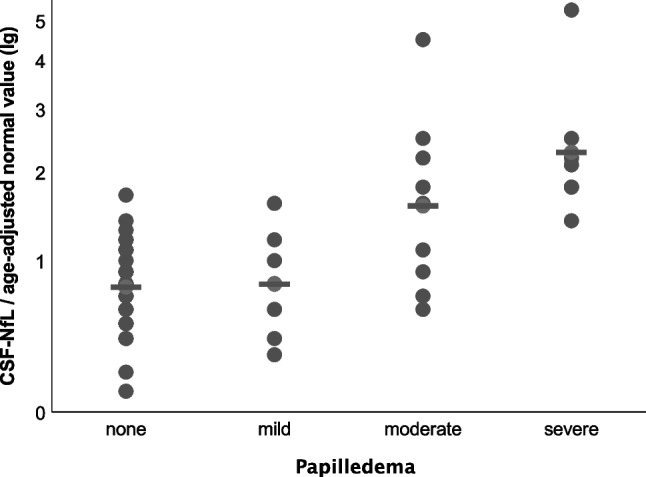
Grouped dot plot comparing the individual CSF-NfL ratio (CSF-NfL/age-adjusted normal value) in all subjects depending on the grade of papilledema. Each dot indicates the CSF-NfL ratio per individual patient. Dashes indicate mean values for each group

**Fig. 4:**
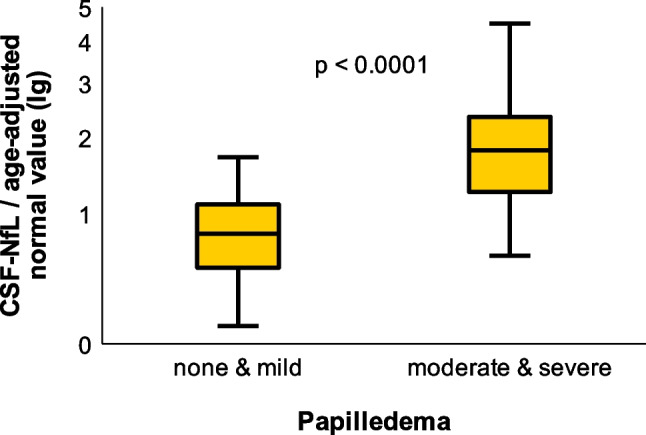
Two box-whisker plots comparing CSF-NfL ratios between the two subgroups of patients with no or with mild papilledema (group 1, *n* = 24; 7) and in patients with moderate or severe papilledema (group 2, *n* = 9; 7). The first group (none and mild papilledema) had a mean CSF-NfL ratio of 0.8 ± 0.4. The group with moderate and severe papilledema showed a higher mean CSF-NfL ratio of 2.1 ± 1.3. Unpaired *t*-test showed a significant difference regarding CSF-NfL ratios between the two groups (i.e., *p* < 0.0001)

CSF-NfL ratios in IIH/IIHWOP-patients were significantly higher than in healthy controls (Table 1, *p* = 0.03, unpaired *t*-test). CSF-NfL ratios rose with the degree of papilledema (Fig. 3). All patients without or with mild papilledema had a mean CSF-NfL ratio of 0.8 ± 0.4, while patients with moderate and severe papilledema showed a higher mean CSF-NfL ratio of 2.1 ± 1.3 (Fig. 4, *p* < 0.0001, unpaired *t*-test). The two groups (group 1: no and mild papilledema vs. group 2: moderate and severe papilledema) did not significantly differ with respect to age and sex. In 10 IIH patients, papilledema had already resolved at the time of examination. In this group, CSF-NfL ratios were lower when compared to IIH patients with papilledema at the time of examination (papilledema, *n* = 23, mean CSF-NfL ratio = 1.7; no papilledema, *n* = 10, mean CSF-NfL ratio = 0.9, *p* = 0.02, unpaired t-test).

This study included two patients with IIHWOP (no papilledema, but with abducens nerve palsy and with ICP of 25 and 31 cmH_2_O, respectively). In one patient, CSF-NfL was normal; in the other patient, it showed a mild, 1.3-fold, increase. Three other patients had sixth nerve palsy and moderate (*n* = 2) or severe (*n* = 1) papilledema, all with increased CSF-NfL ratios (i.e., 4.5, 2.2, 1.8).

## Discussion

In line with a prior study [16], we found a positive correlation of CSF-NfL with the grade of papilledema. IIH patients without papilledema or with resolved papilledema showed no increase of CSF-NfL. Further, CSF-NfL ratios correlated with CSF opening pressure. Thus, we conclude that increased CSF-NfL may herald damage to the optic nerve due to moderate or severe papilledema in IIH. Based on these findings, we assume that papilledema represents the single complication in IIH with potentially permanent axonal damage.

We can only hypothesize concerning the pathophysiological mechanism underlying CSF-NfL release in IIH, but the association of CSF-NfL with both papilledema and CSF opening pressure seems to imply that it results from pressure-induced axonal damage. It still remains unknown whether CSF-NfL in IIH is due to optic nerve damage alone or whether it reflects ubiquitous axonal damage caused by raised intracranial pressure. However, in this study, IIH patients with resolved papilledema had normal CSF-NfL. Moreover, none of our healthy controls showed raised CSF-NfL, despite some of them having slightly increased CSF opening pressures, a finding also reported by Beier et al. [16]. Our study included 5 patients with abducens nerve palsy, some of whom had elevated CSF-NfL, although papilledema was also present in most of them. Our study cohort was too small to investigate whether abducens nerve palsy in IIH also leads to significant CSF-NfL release.

As mentioned above, CSF-NfL has been explored in other neurological disorders, with comparatively high CSF-NfL in inflammatory disorders of the central nervous system. CSF-NfL in inflammatory optic neuritis shows much higher levels than our group of IIH patients [18]. Unlike papilledema in IIH, inflammatory optic neuritis is characterized by a relapsing course with more fulminant axonal damage, which may explain the difference in CSF-NfL levels.

A major limitation of our study is the small cohort and its retrospective nature. More detailed ophthalmological findings, such as perimetric testing and the dependence of CSF-NfL on retinal nerve fibre layer thickness, would be desirable. Moreover, our study lacks correlation with functional visual outcomes and can, therefore, only prepare the ground for future prospective studies. A selection bias may have caused more severely affected IIH patients to be part of this study because only patients who had received a lumbar puncture with CSF-NfL measurement and ophthalmological examination were included. Seemingly less affected patients would not have received such extensive workup.

## Conclusion

Summarizing, our study confirms the relationship between CSF-NfL concentrations and severe papilledema in IIH patients. Furthermore, we report that CSF-NfL rises with the grade of papilledema. In addition, a correlation of CSF-NfL with CSF opening pressure was observed. We conclude that CSF-NfL may represent pressure-induced axonal damage to the optic nerve in IIH, making it a biomarker for optic nerve damage.

Further studies will need to assess the temporal connection of CSF-NfL release with optic nerve damage, papilledema and CSF opening pressure. Depending on the blood assay being used, the correlation between CSF-NfL and serum NfL has been reported to be strong [19]. In the future, serum NfL could be a less invasive biomarker to monitor the disease activity and response to therapy.
